# Mixed primary and secondary vaginal calculi: a case report

**DOI:** 10.1093/jscr/rjad486

**Published:** 2023-10-05

**Authors:** Ghassan Alhajress, Nasser Albqami, Ahmed I Nazer, Omar B Alfraidi, Faisal Khalid Balaraj, Abdullah Alsaghyir, Yahya Ghazwani

**Affiliations:** Division of Urology, Department of Surgery, Ministry of National Guard - Health Affairs, Riyadh 11426, Saudi Arabia; Department of Medicine, King Abdullah International Medical Research Center, Riyadh 11481, Saudi Arabia; Division of Urology, Department of Surgery, Ministry of National Guard - Health Affairs, Riyadh 11426, Saudi Arabia; Department of Medicine, King Abdullah International Medical Research Center, Riyadh 11481, Saudi Arabia; College of Medicine, King Saud bin Abdulaziz University for Health Sciences, Riyadh 14611, Saudi Arabia; Division of Urology, Department of Surgery, Ministry of National Guard - Health Affairs, Riyadh 11426, Saudi Arabia; Department of Medicine, King Abdullah International Medical Research Center, Riyadh 11481, Saudi Arabia; Division of Urology, Department of Surgery, Ministry of National Guard - Health Affairs, Riyadh 11426, Saudi Arabia; Department of Medicine, King Abdullah International Medical Research Center, Riyadh 11481, Saudi Arabia; Division of Urology, Department of Surgery, Ministry of National Guard - Health Affairs, Riyadh 11426, Saudi Arabia; Department of Medicine, King Abdullah International Medical Research Center, Riyadh 11481, Saudi Arabia; Division of Urology, Department of Surgery, Ministry of National Guard - Health Affairs, Riyadh 11426, Saudi Arabia; Department of Medicine, King Abdullah International Medical Research Center, Riyadh 11481, Saudi Arabia; Division of Urology, Department of Surgery, Ministry of National Guard - Health Affairs, Riyadh 11426, Saudi Arabia; Department of Medicine, King Abdullah International Medical Research Center, Riyadh 11481, Saudi Arabia; College of Medicine, King Saud bin Abdulaziz University for Health Sciences, Riyadh 14611, Saudi Arabia

**Keywords:** vaginal calculi, vaginal stone, vesicovaginal fistula

## Abstract

Vaginal calculi are classified according to the pathogenesis of calculus formation. Primary and secondary vaginal calculi. In this article, we present an interesting case that we believe to be a mixed primary and secondary vaginal calculi in which both originated as a result of a vesicovaginal fistula (VVF) and stitches found in the vagina. Although vaginal calculi are a rare disease, a high index of suspicion is needed, especially in patients with a history of gynecological procedures. Since the high recurrence rate of VVF along with its complications, more frequent follow-up and physical examination are required to avoid the recurrence of the disease.

## Introduction

Vaginal calculi are rare entities and due to its low incidence, it might be misdiagnosed [1]. Vaginal calculi are classified according to the pathogenesis of calculus formation [1, 2]. Primary vaginal calculi which are believed to arise as a consequence of stagnating urinary flow accompanied by infected urine within the vagina [1–3]. Thereafter, the calcification of inorganic salt within the urine will gradually result in calculus formation [1, 2]. These abnormalities mainly attributed to genitourinary fistula, dysplasia, and trauma [1, 2]. Whereas the development of secondary vaginal calculi is mostly due to urinary inorganic salt crystallization around eroded surgical mesh or other foreign body in the vagina [1–3]. Most of the cases reported were primary and fewer were secondary vaginal calculi. However, in this article we present an interesting case that we believe to be a mixed primary and secondary vaginal calculi in which both originated as a result of a vesicovaginal fistula (VVF), and stitches found in the vagina.

## Case report

**Figure 1:**
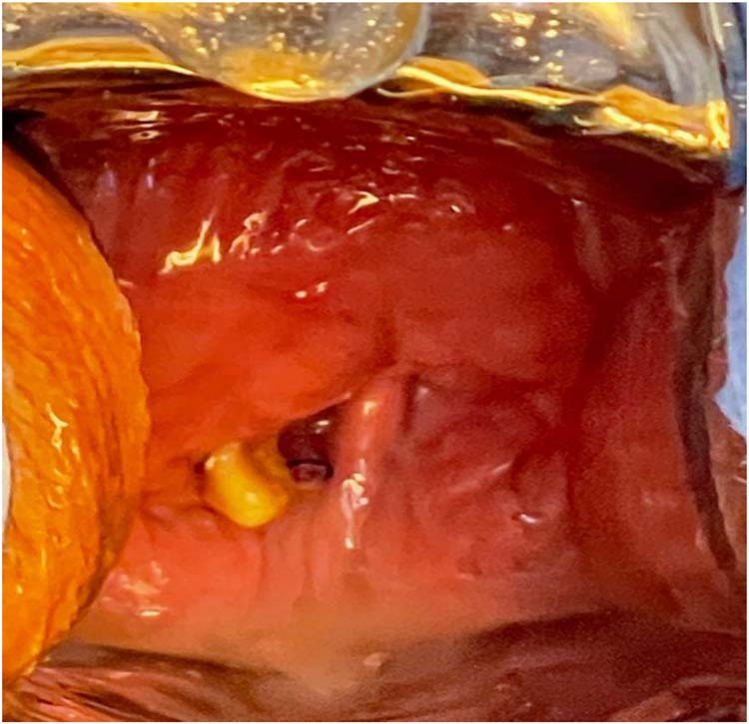
A vaginoscopy showed calcified stitches along with stone.

**Figure 2:**
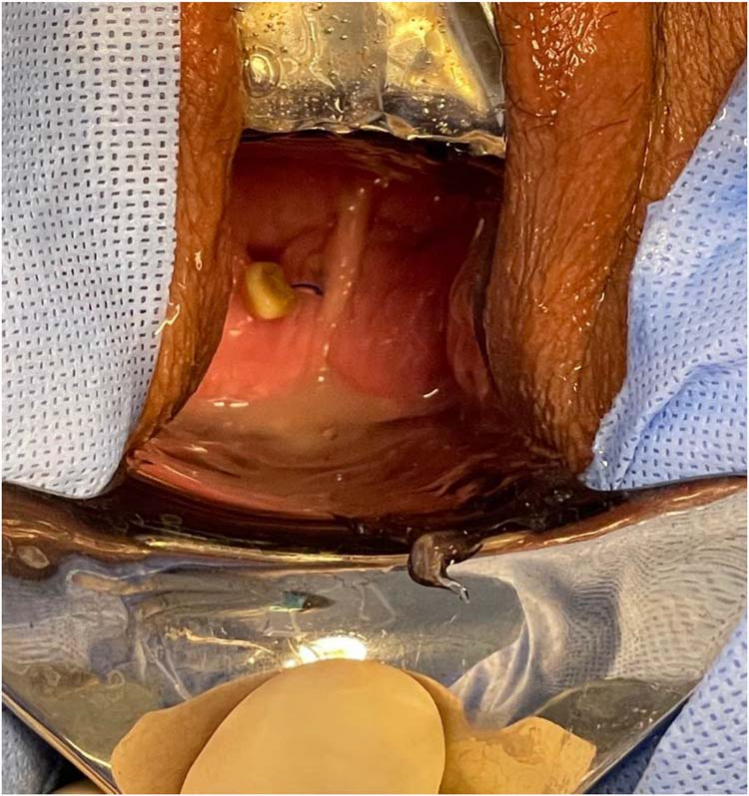
A vaginoscopy showed calcified stitches along with stone.

**Figure 3:**
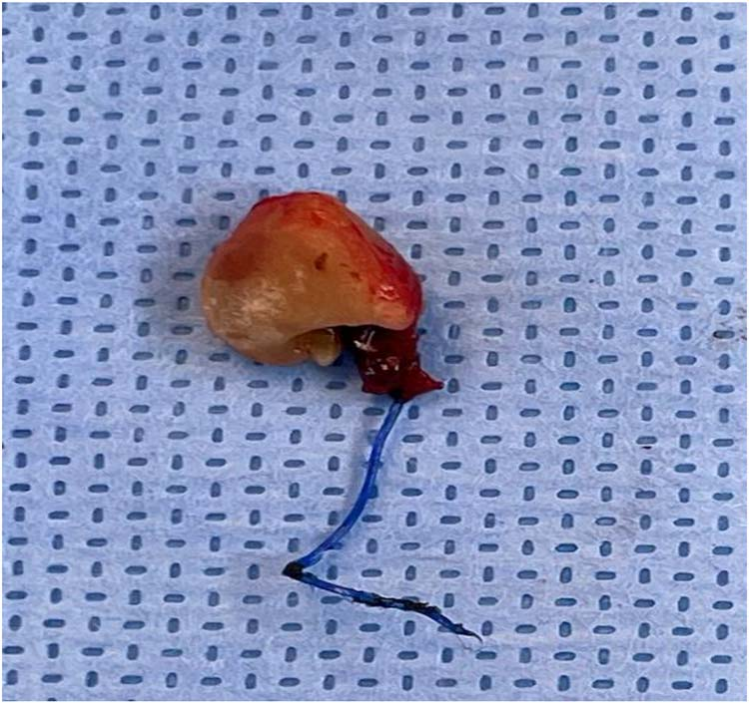
The stone and the stitches after removal.

**Figure 4:**
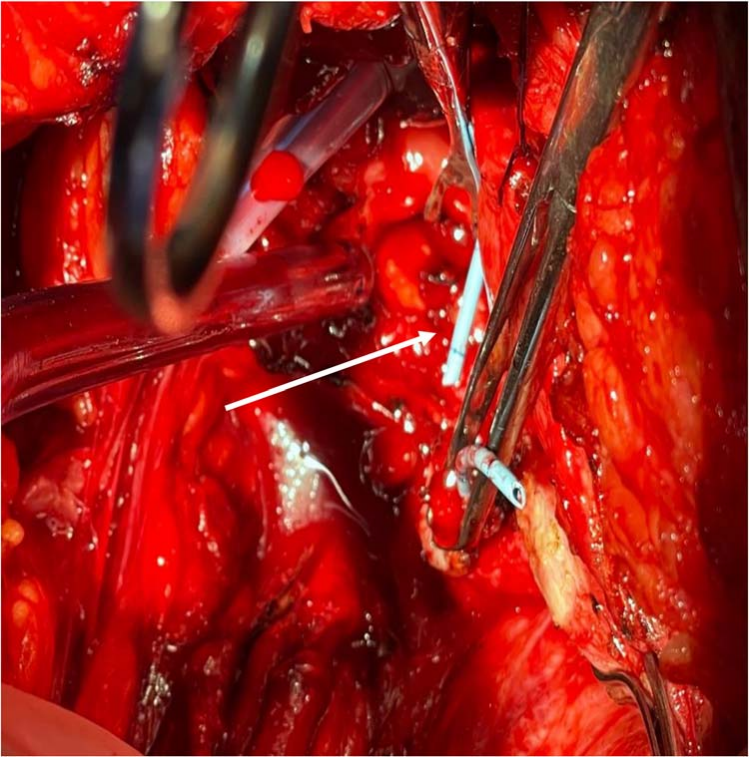
Fistula tract is cannulated with a guidewire as pointed in the arrow.

**Figure 5:**
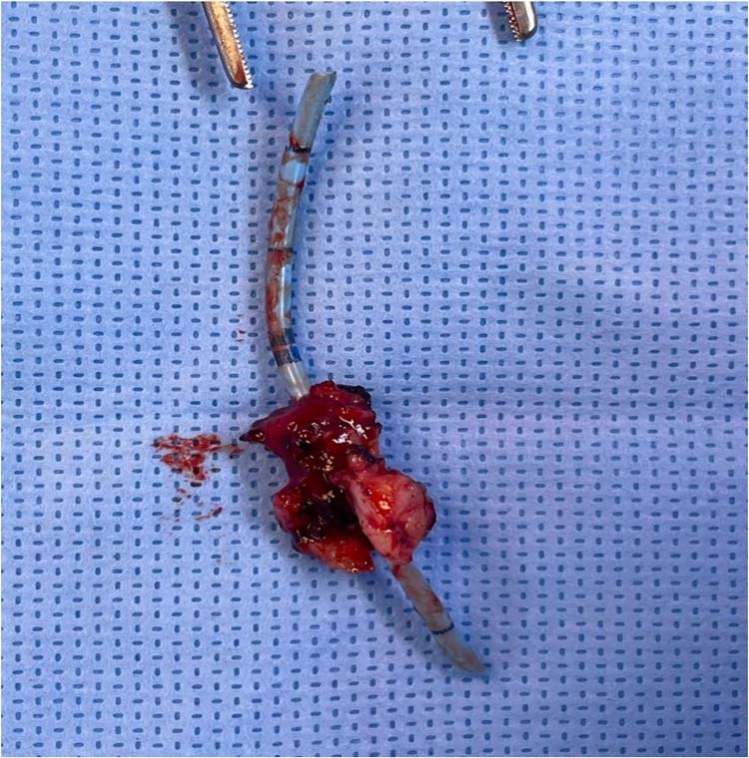
Fistula tract after complete excision.

A 54-year-old female patient with a history of recurrent urinary tract infection (UTI) was admitted electively to the hospital as a case of VVF for repair. Her chief complaints were intermittent lower abdominal pain and urine leakage for ~1 year. Gynecological examination was unremarkable. She had a history of Dysfunctional Uterine Bleeding that was managed eventually by total abdominal hysterectomy with bilateral salpingo-oophorectomy. Unfortunately, bladder injury was confirmed intraoperatively. Since then, the patient started to complain of persistent urinary incontinence and urine coming out of the vagina. A total of 6 weeks later, Computed Tomography (CT) cystography was done, and it confirmed the presence of a low-lying VVF for which the patient underwent first repair. The surgery outcome was undesirable since the patient had a recurrence in ˂1 month. Moreover, postoperative CT cystography confirmed recurrence as it showed a VVF extending between the upper vagina and upper posterior wall of the bladder. After admission, preoperative work-up was done. At that time, the urine culture turned out to be positive for *Escherichia coli*. Therefore, the patient was managed initially with culture sensitive IV antibiotic and foley catheter. The patient was planned for surgery once the urine culture was negative. At the beginning, a diagnostic cystoscopy was performed and revealed highly suspicious areas in the left lateral and posterior wall of the bladder suggesting a fistula. Afterward, a vaginoscopy was done and showed calcified stitches along with stone (Figs 1 and 2). The stone and the stitches were removed (Fig. 3). The fistula tract then was cannulated with a guidewire (Fig. 4). A clear connection was seen between the vagina and the bladder. Afterward, the plan was to proceed with the repair. Lower midline incision was made; extensive adhesion was noted after opening the peritoneum. Despite the adhesion, bladder dissection was successful. The posterior aspect of the bladder wall was dissected and separated from the vagina with extreme difficulty. After that, a longitudinal cystotomy was done and the fistula tract was completely excised (Fig. 5). The vaginal wall was closed using 3-0 Vicryl and the bladder closed in two layers with 2-0 and 3-0 Vicryl. Unfortunately, due to the extensive adhesion, a small (2 cm) in diameter rectal injury occurred due to traction and was managed properly by general surgery. Finally, a drain and a large transurethral catheter were placed. Postoperatively, the patient initially complained of mild generalized abdominal pain and vomiting. The patient had no complaints upon discharge, and the plan was to keep the patient on the foley catheter. A total of 3 weeks later the patient was seen in the clinic, and she underwent a cystourethrogram, which turned out to be unremarkable. The patient had a successful outcome with no sign of recurrence.

## Discussion

Vaginal calculi are rare pathologies in which calcification is primarily a result of urine stasis and infection [1]. Static urine within the vagina can create an ideal environment for urease producing organisms (such as *E. coli*) to grow and multiply [1]. Subsequently, the urease will gradually shift the PH from being Acidic to Alkaline, which facilitates triphosphate crystallization. Up-to-date, triphosphate is considered to be the most common constitute in vaginal calculi [1]. In this patient, the presentation which was highly suggestive for UTI especially when the urine culture turned to be positive for *E. coli*. Vaginal calculi can arise from multiple etiologies including VVF [1]. VVF is most commonly acquired following gynecological surgeries, radiation therapy, or malignancies [1]. Furthermore, VVF can develop secondary to bladder wall necrosis, which is commonly seen after major gynecological procedures [1]. In this case, the patient had a history of total abdominal hysterectomy with bilateral salpingo-oophorectomy that was complicated by bladder injury. Despite the prolonged process by which vaginal calculi form secondary to VVF, in this case the patient developed vaginal calculi more rapidly [1]. Given the fact that the patient had a VVF repair 1 month earlier. This acceleration might be explained by the mixed pathogenesis of both primary and secondary vaginal calculi. This disease can be treated surgically by different approaches, including abdominal and transvaginal access [4]. Despite the debate over which approach is superior, the decision should be individualized based on the type of the fistula and the surgeon’s experience [4]. In this case, the patient had a complex fistula since it was the second repair, so the abdominal approach was more suitable. The surgery was performed in two steps: removal of the stones and stitches followed by fistula excision. The surgery was a success with no complications postoperatively.

## Conclusion

Although vaginal calculi are a rare disease, a high index of suspicion is needed, especially in patients with a history of gynecological procedures. A major cause of vaginal calculi formation is genitourinary fistula combined with recurrent infection. Since the high recurrence rate of VVF along with its complications, more frequent follow-up and physical examination are required to avoid the recurrence of the disease.

## Acknowledgement

I would like to acknowledge and give my warmest thanks to co-authors who made this work possible.

## Conflict of interest statement

The authors declare that there is no conflict of interest.

## Funding

None declared.

## Data availability

All date required is mentioned in the manuscript.
